# The Role of [^18^F]FDG PET/CT Prior to and During Neoadjuvant Chemotherapy for Soft Tissue Sarcomas

**DOI:** 10.3390/curroncol32050257

**Published:** 2025-04-28

**Authors:** Stijn J.C. van der Burg, Bernies van der Hiel, Lotte Heimans, J. Martijn Kerst, Michel W.J.M. Wouters, Petur Snaebjornsson, Yvonne M. Schrage, Winette T.A. van der Graaf, Winan J. van Houdt

**Affiliations:** 1 Department of Surgical Oncology, Netherlands Cancer Institute, Antoni van Leeuwenhoek Hospital (NKI-AVL), Plesmanlaan 121, 1066 CX Amsterdam, The Netherlands; s.vd.burg@nki.nl (S.J.C.v.d.B.); m.wouters@nki.nl (M.W.J.M.W.); y.schrage@nki.nl (Y.M.S.); 2 Department of Nuclear Medicine, Netherlands Cancer Institute, Antoni van Leeuwenhoek Hospital (NKI-AVL), Plesmanlaan 121, 1066 CX Amsterdam, The Netherlands; b.vd.hiel@nki.nl; 3 Department of Medical Oncology, Netherlands Cancer Institute, Antoni van Leeuwenhoek Hospital (NKI-AVL), Plesmanlaan 121, 1066 CX Amsterdam, The Netherlands; l.heimans@nki.nl (L.H.); j.kerst@nki.nl (J.M.K.); w.vd.graaf@nki.nl (W.T.A.v.d.G.); 4 Department of Pathology, Netherlands Cancer Institute, Antoni van Leeuwenhoek Hospital (NKI-AVL), Plesmanlaan 121, 1066 CX Amsterdam, The Netherlands; p.snaebjornsson@nki.nl; 5 Faculty of Medicine, University of Iceland, 101 Reykjavik, Iceland

**Keywords:** [^18^F]FDG PET/CT, neoadjuvant chemotherapy, soft tissue sarcoma, pathologic response, disease recurrence

## Abstract

This retrospective, single-center study investigates the association between PET parameters and pathological response or disease recurrence in patients with soft tissue sarcoma (STS) treated with neoadjuvant chemotherapy (NACT). The maximum standardized uptake value (SUVmax_BL_), metabolic tumor volume (MTV_BL_), and total lesion glycolysis (TLG_BL_) were measured at baseline [^18^F]FDG PET/CT and the change in percentage (ΔSUVmax, ΔMTV, ΔTLG) from baseline to early evaluation [^18^F]FDG PET/CT was calculated. The optimal cutoff values of the different PET parameters for pathological response, defined as <10% residual viable tumor (RVT) or >15% fibrosis/hyalinization, and recurrence-free survival were obtained for analysis. Forty-two patients who underwent baseline [^18^F]FDG PET/CT and NACT followed by surgery were included between January 2015 and January 2023. The primary diagnoses were angiosarcoma (n = 15), leiomyosarcoma (n = 15), sarcoma not otherwise specified (n = 9) and synovial sarcoma (n = 3). Twenty-eight (66.6%) patients underwent an early evaluation PET/CT. MTV_BL_, TLG_BL_, and ΔSUVmax (*p* = 0.024; *p* = 0.042, *p* = 0.009, respectively) values above the cutoff were associated with a pathological response based on RVT. ΔSUVmax, ΔMTV, and ΔTLG (*p* = 0.002; *p* = 0.019; *p* = 0.039, respectively) values above the cutoff were positively related to >15% fibrosis/hyalinization. MTV_BL_, TLG_BL,_ and ΔMTV (*p* = 0.014; *p* = 0.022; *p* = 0.034, respectively) values above the cutoff were prognostic for the recurrence of disease. [^18^F]FDG PET/CT has a promising role in STS patients treated with NACT.

## 1. Introduction

Soft tissue sarcomas (STSs) are a heterogeneous and rare group of cancers originating from mesenchymal tissue [1,2] with an incidence of approximately 4–5 per 100,000 per year in Europe [3]. The cornerstone of treatment for local STSs is the resection of the tumor, often supplemented by preoperative or postoperative radiotherapy for high-grade STSs to reduce local recurrence rates [4]. While there is no clear consensus on the use of neoadjuvant chemotherapy (NACT), there are two potential indications: (1) it may be employed to reduce tumor size and increase the likelihood of achieving resection with clear margins in borderline resectable tumors, and (2) recent studies utilizing nomograms have demonstrated that a potential survival benefit is associated with NACT in high-risk sarcomas [5,6].

During NACT, early evaluation imaging scans can be performed to assess tumor progression or response. In case of significant tumor progression, one might move the planned surgery forward to prevent losing the window of opportunity for resecting the tumor with clear margins. Furthermore, in case of a clear response, additional cycles of potentially toxic chemotherapy could be avoided in patients unlikely to benefit from further treatment. Depending on the location of the STS, computed tomography (CT) or magnetic resonance imaging (MRI) is typically used to evaluate the extent of the disease. However, the role of these imaging modalities in the early evaluation of treatment response remains controversial, as their findings show limited correlation with the pathologic response and clinical outcomes [7]. Therefore, to support treatment decision-making, there is a need for improved early evaluation tools to predict the pathologic response and clinical outcomes.

18F-fluorodeoxyglucose positron emission tomography/computed positron emission tomography/computed tomography ([^18^F]FDG PET/CT) is a non-invasive imaging modality that visualizes glucose metabolism. In STS, increased glucose metabolism is associated with the high-grade nature of the tumor [8,9] and with worse survival [10], suggesting that and [^18^F]FDG PET/CT could play a role in response prediction and the early evaluation of STS patients undergoing NACT. However, robust evidence supporting this hypothesis is limited [8,11,12,13,14].

In our center, to address the limitations associated with CT, we initiated the use of [^18^F]FDG PET/CT imaging in patients with STS undergoing NACT. This study evaluates the utility of baseline and early evaluation [^18^F]FDG PET/CT in these patients. The primary objective is to explore the association between [^18^F]FDG PET/CT using various PET parameters and the pathological response, while the secondary objective is to evaluate the relationship between PET parameters and recurrence-free survival.

## 2. Methods

### 2.1. Patients

In this single-center study, we retrospectively included all patients with a confirmed diagnosis of STS between January 2015 and January 2023, who received NACT either due to a high-risk tumor or to reduce tumor size, thereby increasing the likelihood of achieving resection with clear margins. The included patients were required to have undergone at least a baseline [^18^F]FDG PET/CT scan and subsequent tumor resection following neoadjuvant treatment. Gastrointestinal stromal tumors were excluded. Patients presenting with local recurrences or metastases were included in the analysis of the pathological response, but excluded from survival analyses. Data regarding the chemotherapy regime, number of treatment cycles, and the use of additional radiotherapy were collected from electronic patient records. The study was approved by the local ethical committee (IRBd23-280).

### 2.2. [^18^F]FDG PET/CT Protocol

[^18^F]FDG PET/CT scans were performed on a cross-calibrated EARL-accredited Philips Gemini TF time-of-flight 16 or Philips Gemini TF big-bore PET/CT scanner (Philips, Cleveland, OH, USA). Patients had adequate fluid intake and fasted for at least 4 h prior to the intravenous administration of 3.5 MBq/kg [^18^F]FDG. Approximately 60 min after injection, PET/CT scans were acquired from the base of the skull to the thighs at 2–4 min per bed position in a supine position, according to the ‘European Association of Nuclear Medicine (EANM) guideline for oncology 18F-FDG PET/CT imaging’ [15,16]. To enable the quantitative evaluation of the different scanners, image reconstruction was performed according to EARL standard 1 [17,18].

### 2.3. PET Parameters, Pathological Outcomes, and Oncological Outcome

Tumor uptake on [^18^F]FDG PET/CT was assessed both visually and semi-quantitatively prior to and, if available, after 1 to 3 cycles of NACT. The tumor masses were automatically delineated with a fixed standard uptake value (SUV) threshold of 2.5, resulting in a region of interest (ROI) of the summed lesions [19]. The ROI was inspected visually and manually corrected if necessary. On each scan, the maximum standardized uptake value (SUVmax), metabolic tumor volume (MTV) and total lesion glycolysis (TLG) (=MTV*SUVmean) of the summed lesions were calculated. On evaluation scans, the percentage difference compared to the baseline of the different PET parameters was calculated. Baseline (SUVmax_BL_, MTV_BL_, TLG_BL_) and follow-up (ΔSUVmax, ΔMTV, ΔTLG) PET parameters were used for further analysis. All pathology samples were reviewed by a specialized sarcoma pathologist according to the World Health Organization’s classification of soft tissue tumors [20]. Representative sections were obtained from each tumor and the assessed percentage of residual viable tumor (RVT), i.e., the percentage of viable tumor cells, and fibrosis/hyalinization were used for the purpose of this study. Pathological response was defined as <10% RVT or >15% fibrosis/hyalinization, based on recent literature [21,22,23,24]. As the oncological outcome, recurrence-free survival was used, which includes both local and distant recurrences.

### 2.4. Statistical Analysis

In the case of continuous data, depending on the distribution of the dataset, either the mean with standard deviation (SD) or the median with interquartile range (IQR) was reported. The normality of the distributions was assessed using the Shapiro–Wilk test. Categorical data were presented as frequencies and percentages. The means between histologies were compared using one-way ANOVA and medians with the Kruskal–Wallis test. Receiver operating characteristic (ROC) curves were constructed to ascertain the optimal cutoff values for SUVmax_BL_, MTV_BL_, TLG_BL_, ΔSUVmax, ΔMTV, and ΔTLG, with the objective of predicting the pathological response and recurrence-free survival (RFS). In order to be included for further analysis, variables were required to have an area under the curve (AUC) greater than 0.7, indicating at least a fair predictive value [25]. The optimal cutoff values were determined using Youden’s index [26]. Following that, the included continuous variables were dichotomized and the association with a pathological response and the oncological outcomes was analyzed using binary logistic regression and Cox regression analyses. To explore the association between pathological and oncological outcomes, the log-rank test was used.

## 3. Results

### 3.1. Patient, Tumor, Treatment, and [^18^F]FDG PET/CT Scan Characteristics

Between January 2015 and January 2023, 44 patients with STS underwent a baseline [^18^F]FDG PET/CT scan and NACT prior to surgery. Two patients were excluded from analysis, one due to a changed histology after resection (angiosarcoma became telangiectatic osteosarcoma) and one due to metabolic inactivity (n = 1), resulting in 42 evaluable patients. The median age was 60 years (IQR: 51–66 years), with 27 females (64%) and 15 males (36%). The most common histological tumor types were angiosarcoma (AS) and leiomyosarcoma (LMS), both present in 15 patients (36%). All patient, tumor, and treatment characteristics are displayed in Table 1. The median time between the baseline [^18^F]FDG PET/CT scan and the initiation of NACT was 11 days (IQR 6–27 days).

In 28 (67%) patients, an additional evaluation [^18^F]FDG PET/CT scan was performed. The majority (n = 21, 75%) underwent [^18^F]FDG PET/CT after two cycles of chemotherapy. In the remaining seven patients, [^18^F]FDG PET/CT was performed after one (n = 3, 11%) or three (n = 4, 14%) cycles of chemotherapy. The patient, tumor and treatment characteristics of this subgroup are displayed in Supplementary Table S1. For survival data, only the 35 patients who were treated for a primary tumor were included. The median follow-up time was 27 months (IQR 16–41 months); 13 (39.4%) patients had disease recurrence and 10 (30%) patients died.

### 3.2. PET Parameters and Pathological Response per Histological Tumor Type

**Figure 1:**
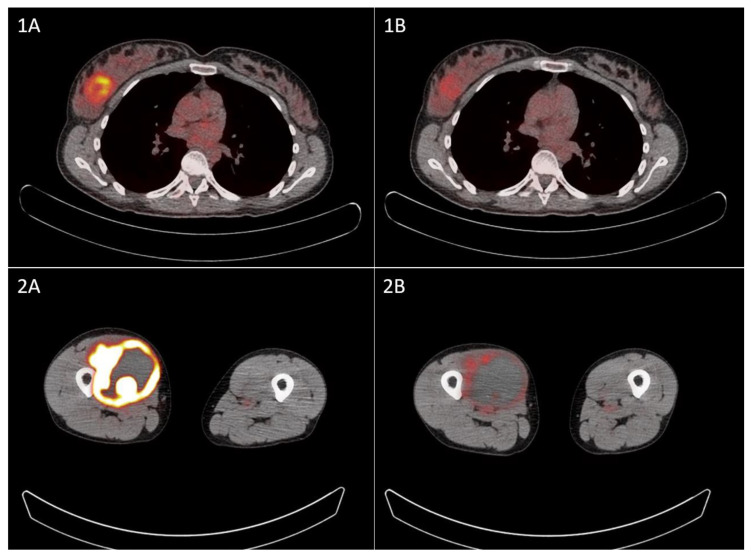
Baseline [^18^F]FDG PET/CT of an angiosarcoma of the breast (**1A**) and after one cycle of paclitaxel (**1B**), and the baseline [^18^F]FDG PET/CT of sarcoma NOS in the right upper leg (**2A**) and after 2 cycles of doxorubicine/ifosfamide (**2B**).

On baseline [^18^F]FDG PET/CT, there was a significant difference in MTV_BL_ (*p* < 0.001) and TLG_BL_ (*p* = 0.001) between the four histological tumor types, with the highest median MTV_BL_ and TLG_BL_ for sarcoma NOS and the lowest for AS (Table 2). There was no significant difference in SUVmax_BL_ nor in ΔSUVmax, ΔMTV, or ΔTLG, although the ΔSUVmax of AS was higher compared to the other histologic subtypes. Figure 1 demonstrates [^18^F]FDG uptake before and after NACT in two patients. The pathological response rate differed significantly between the histological tumor types for RVT (*p* = 0.018), but not for fibrosis/hyalinization (*p* = 0.373). The highest pathological response rate based on RVT was seen in sarcoma NOS (68%) and was lowest in LMS (7%). All PET parameters and pathological response rates are displayed in Table 2.

A sub-analysis with log rank tests demonstrated a significantly better RFS for patients with a pathologic response based on both RVT (*p* = 0.024) and fibrosis/hyalinization (*p* = 0.034).

### 3.3. Predictive Performance of PET Parameters

Area under the curve (AUC) analyses for all six PET parameters (SUVmax_BL_, MTV_BL_, TLG_BL_, ΔSUVmax, ΔMTV, ΔTLG) with the three outcome variables (pathological response based on RVT or fibrosis/hyalinization, and recurrence) were conducted to evaluate the predictive performance. Only PET parameters with an AUC > 0.7 were taken into account for further analysis. For pathologic response (RVT, fibrosis/hyalinization or both), an AUC > 0.7 was found for MTV_BL_, TLG_BL_ and not SUV max, and all three evaluation ΔPET parameters. For recurrence, MTV_BL_, TLG_BL_ and ΔMTV had an AUC > 0.7. Table 3 shows all AUC values with the corresponding optimal cutoff values.

### 3.4. Univariable Regression Analysis with Optimal Cutoffs for Baseline PET Parameters

**Figure 2:**
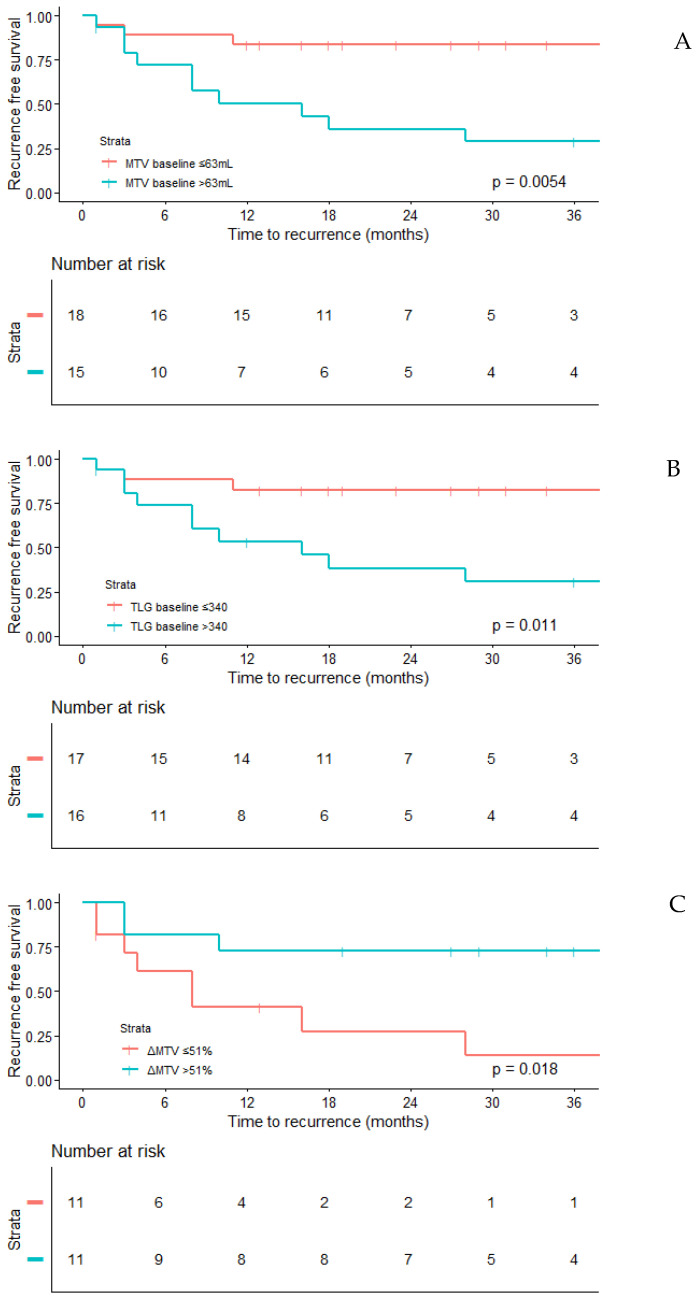
Kaplan–Meier curves for recurrence-free survival per MTV_BL_ (**A**), TLG_BL_ (**B**) and ΔMTV (**C**).

When using the optimal cutoffs for the three different outcome variables, MTV_BL_ and TLG_BL_ above the cutoff were both negatively associated with a pathological response based on RVT (MTV_BL_: OR 0.20, 95%CI 0.05–0.82, *p* = 0.024, TLG_BL_: OR 0.25, 95%CI 0.07–0.95, *p* = 0.042) and positively associated with recurrence (MTV_BL_: HR 5.11, 95%CI 1.40–18.67, *p* = 0.014, TLG_BL_: HR 4.56, 95%CI 1.25–16.64, *p* = 0.022) (Table 4 and Figure 2A,B). None of the baseline PET parameters revealed significant associations with a pathological response based on fibrosis/hyalinization.

### 3.5. Univariable Regression Analysis with Optimal Cutoffs for Change in PET Parameters

During NACT, an ΔSUVmax value above the cutoff was positively associated with a pathologic response based both on RVT (OR 42.00, 95%CI 3.76–469.01, *p* = 0.002) and fibrosis/hyalinization (OR 42.00, 95%CI 3.76–469.01, *p* = 0.002), while ΔMTV and ΔTLG were only associated with fibrosis/hyalinization (ΔMTV: OR 11.67, 95%CI 1.49–91.54, *p* = 0.019, ΔTLG: OR 8.13, 95%CI 1.12–59.21, *p* = 0.039). Only ΔMTV was associated with RFS, with significantly fewer recurrences in patients with an ΔMTV value above the cutoff (HR 0.23, 95%CI 0.06–0.90, *p* = 0.034) (Table 4 and Figure 2C).

## 4. Discussion

The present study evaluated the utility of baseline and early evaluation [^18^F]FDG PET/CT as a predictive imaging tool for patients who underwent [^18^F]FDG PET/CT as part of their treatment with NACT for STS in our institution. We found that the PET parameters MVT_BL_, TLG_BL_, and ΔSUVmax were associated with a response based on RVT, and ΔSUVmax, ΔMTV and ΔTLG were associated with a pathological response based on fibrosis/hyalinization. The PET parameters MTV_BL_, TLG_BL_ and ΔMTV were associated with disease recurrence.

A number of other studies have investigated the role of [^18^F]FDG PET/CT at baseline or as an early evaluation during or after NACT in patients with STS [8,12,13,14,27,28,29,30]. However, the included histological tumor types and analyzed PET parameters differ between these studies. Furthermore, the endpoints in these studies vary, from association with different definitions of pathological response to association with survival outcomes. Due to the heterogeneity of these studies, a standard protocol for the use of [^18^F]FDG PET/CT in patients receiving NACT for STS has not yet been established.

In four studies evaluating the prognostic value of baseline PET parameters in a patient population without NACT but with upfront surgery, high SUVmax was a prognostic factor for worse OS and RFS [8,13,14,28]. This association was not observed in our study, which may be attributed to the variety of tumor grades included in these studies. These studies included tumors across all grades, whereas our study focused exclusively on the histological tumor types eligible for NACT, which were predominantly high-grade. This difference suggests that SUVmax may have less prognostic value in a cohort consisting mainly of high-grade tumors. However, the lacking significance in our cohort could also be attributed to the low number of patients included for survival analysis.

Three out of the four studies mentioned above also investigated volume-based PET parameters at baseline as a prognostic biomarker for survival [8,13,14]. Chen et al. performed a meta-analysis and found a significant association between MTV and both OS and RFS based on three studies, while only one study was reported a significant association between TLG and both OS and RFS [14]. Reyes Marles et al. only found an association between MTV and TLG with OS [8], while Hong et al. did not find any prognostic value for MTV or TLG [13]. In our study, MTV_BL_ and TLG_BL_ were a prognostic factor for RFS, but not for OS. These discrepancies across studies are likely due to heterogeneity in tumor and treatment characteristics, highlighting the need for larger cohorts to clarify the prognostic role of baseline volume-based PET parameters.

To our knowledge, no previous studies have analyzed the predictive value of baseline [^18^F]FDG PET/CT for pathological response. Our study showed a significant association between MTV_BL_ and TLG_BL_ with a pathological response based on RVT. Our results indicate for the first time that baseline volume-based PET parameters might predict a pathological response, although larger series are obviously needed to validate these findings. If confirmed in future studies, this could have significant clinical implications and be of great importance, since it could help in selecting patients that could benefit from NACT.

When evaluating [^18^F]FDG PET/CT as a predictive tool for early response assessment, three studies investigated SUVmax after the first cycle of NACT, but no studies that incorporated volume-based parameters were found. Benz et al. demonstrated a significant association between a larger decrease in SUVmax and achieving a pathological response based on >95% necrosis [11]. In contrast, in the study of Tateishi et al., no association with an early reduction in SUVmax and a pathologic response defined as ≤10% RVT was found [12]. Herrmann et al. reported from their multivariable regression analysis an association between a large reduction in SUVpeak and survival [29]. In our cohort, a larger reduction in SUVmax was associated with a higher chance of a pathological response, based on both RVT and fibrosis/hyalinization. Strong early reductions in MTV and TLG were also associated with a pathological response based on fibrosis/hyalinization and with a recurrence of disease, which to our knowledge are all novel findings. These findings promote the [^18^F]FDG PET/CT scan as an early evaluation method to aid in deciding whether to continue NACT for patients with STS.

Although RVT remains the most commonly used biomarker for determining pathological response in STS, there has been growing interest in fibrosis/hyalinization as potentially superior pathological biomarkers for assessing response following NACT [21,22,23]. In our cohort, a reduction in all three PET parameters was significantly associated with a pathological response based on fibrosis/hyalinization. Conversely, the baseline volumetric PET parameters were associated with a pathological response based on RVT. A sub-analysis of the prognostic value of the pathological response showed that a pathologic response based both on RVT and fibrosis/hyalinization was associated with the recurrence of disease. Therefore, based on this cohort, neither RVT or fibrosis/hyalinization is superior as a definition of a pathologic response.

It is important to acknowledge the limitations of this study, where we retrospectively evaluated the clinical management of patients with an STS who received NACT either due to borderline resectable tumors or their high risk of recurrence according to nomograms. The retrospective nature and the different indications for NACT resulted in a variation in the histological tumor types, the location of tumors, chemotherapy schedules, the use of neoadjuvant radiotherapy and the timing of the early evaluation scan. In addition, significant differences in MTV_BL_ and TLG_BL_ were observed between histologic tumor types. However, these differences were probably caused by the tumor size rather than the histology. Unfortunately, due to the small sample size, the use of multivariate regression and stratification for the above mentioned possible confounders was not possible. Furthermore, the retrospective design of the study may have introduced selection bias, so correction in a larger cohort is desirable.

Based on our findings, baseline [^18^F]FDG PET/CT may help in identifying STS patients who would benefit from NACT by predicting their pathologic response. In addition, early evaluation [^18^F]FDG PET/CT could complement CT and MRI in predicting pathologic response. Having this additional information could support the decision-making process for administering NACT to specific patients, both within the multidisciplinary tumor board and in discussions with patients. However, the limitations of this study are too substantial to draw definite conclusions. To clarify the role of [^18^F]FDG PET/CT in this patient population, larger prospective studies are needed, with a focus on the optimal timing of [^18^F]FDG PET/CT and the corresponding histology-specific cutoff value. Currently, the STRASS 2 trial, a randomized, phase 3 trial comparing neoadjuvant chemotherapy and surgery to surgery alone, is open to determine the role of NACT for high-grade retroperitoneal DDLPS and LMS [31]. In a sub-study of this trial, the utility of [^18^F]FDG PET/CT will be examined as well. To determine the role of [^18^F]FDG PET/CT scans in patients with extremity and trunk wall STS treated with NACT, new larger studies need to be initiated.

In addition to the qualitative PET parameters evaluated in this study (SUVmax, MTV, TLG), the application of radiomics in predicting the response to NACT for STS is an emerging area of research [32,33,34,35,36]. Radiomics involves the extraction of quantitative features from imaging data, such as MRI, CT, or [^18^F]FDG PET/CT, including gray-level patterns and dynamic characteristics such as tracer uptake rates. With the introduction of radiomics, the ability to predict responders and non-responders may improve even further, potentially further reducing the use of toxic chemotherapy in patients who are unlikely to benefit.

## 5. Conclusions

With the result of this study, we underscore the potential utility of baseline and early evaluation [^18^F]FDG PET/CT for making treatment decisions in patients with STS treated with NACT, allowing for more personalized patient selection or better guidance during treatment to avoid unnecessary toxic side effects without achieving a response. However, our findings have to be confirmed in larger prospective studies.

## Figures and Tables

**Table 1:** Patient, tumor, and treatment characteristics for all patients per histologic subtype.

	All Patients		AS		LMS		Sarcoma NOS		SS	
	N = 42		N = 15		N = 15		N = 9		N = 3	
Sex										
Male	15	(36)	3	(20)	6	(40)	4	(44)	2	(67)
Female	27	(64)	12	(80)	9	(60)	5	(56)	1	(33)
Age (IQR)	60	(51–66)	66	(55–74)	60	(52–62)	53	(49–60)	47	-
Stage										
Primary	35	(84)	11	(73)	12	(80)	7	(78)	3	(100)
Recurrence	6	(14)	3	(20)	3	(20)	2	(22)	0	-
Metastasis	1	(2)	1	(7)	0	-	0	0	0	-
FNCLCC grade										
1	5	(12)	0	-	5	(33)	0	-	0	-
2	10	(24)	2	(13)	7	(47)	1	(11)	0	-
3	13	(31)	2	(13)	3	(20)	8	(89)	0	-
Not graded ^a^	14	(33)	11	(74)	0	-	0	-	3	(100)
Location										
Extremity	13	(31)	3	(20)	1	(7)	6	(67)	3	(100)
Mamma	10	(24)	10	(67)	0	-	0	-	0	-
Retroperitoneal	8	(19)	0	-	8	(53)	0	-	0	-
Abdomen	5	(12)	0	-	5	(33)	0	-	0	-
Trunk wall	3	(7)	0	-	1	(7)	2	(22)	0	-
Other	3	(7)	2	(13)	0	-	1	(11)	0	-
Preoperative RT										
Yes	9	(21)	2	(13)	2	(13)	3	(33)	2	(67)
No	33	(79)	13	(87)	13	(87)	6	(67)	1	(33)
Chemotherapy										
Paclitaxel	14	(33)	15	(93)	0	-	0	-	0	-
Dox/DTIC	14	(33)	0	-	14	(93)	0	-	0	-
Dox/ifos	13	(31)	0	-	1	(7)	9	(100)	3	(100)
TAC	1	(2)	1	(7)	0	-	0	-	0	-
N of cycles										
3	13	(31)	3	(20)	4	(27)	5	(56)	1	(33)
4	21	(50)	8	(53)	7	(47)	4	(44)	2	(67)
5	1	(2)	0	-	1	(7)	0	-	0	-
6	7	(17)	4	(27)	3	(20)	0	-	0	-
Early evaluation scan										
After 1 cycle	3	(7)	1	(7)	1	(7)	1	(11)	0	-
After 2 cycles	21	(50)	4	(27)	10	(67)	4	(45)	3	(100)
After 3 cycles	4	(10)	2	(13)	0	-	2	(22)	0	-
No	14	(33)	8	(53)	4	(27)	2	(22)	0	-

Values are n (%) unless otherwise indicated. Abbreviations: AS = angiosarcoma, LMS = leiomyosarcoma, Sarcoma NOS = Sarcoma not otherwise specified, SS = synovial sarcoma, N = Number, IQR = inter quartile range, FNCLCC = Fédération Nationale des Centres de Lutte Contre le Cancer, RT = radiotherapy, Dox/ifos = doxorubicine/ifosfamide, Dox/DTIC = doxorubicine/dacarbazine, TAC = docetaxel, doxorubicin, cyclophosphamide, ^a^ = pathologist in our center do not routinely grade angiosarcoma and synovial sarcoma since they are always considered as high grade.

**Table 2:** [^18^F]FDG PET parameters and pathological response per histological subtype.

	AS	LMS	Sarcoma NOS	SS	*p*
Baseline [^18^F]FDG PET/CT, (N=)	15	15	9	3	
^a^ SUVmaxBL	13.6 (5.1–20.8)	11.4 (6.7–26.8)	19.6 (135–30.0)	8.2	0.137
^a^ MTVBL	13 (2–78)	275 (64–358)	3421 (116–996)	35	<0.001 *
^a^ TLGBL	100 (11–365)	1185 (195–3167)	1367 (699–5846)	119	0.001 *
Evaluation [^18^F]FDG PET/CT, (N=)	7	11	7	3	
^b^ ΔSUVmax	61 (28)	35 (11)	44 (36)	31 (11)	0.145
^b^ ΔMTV	42 (27)	51 (33)	51 (30)	36 (25)	0.887
Missing	2 (28.6)	0	1 (14.3)	1 (33)	
^b^ ΔTLG	55 (32)	61 (30)	57 (33)	51 (13)	0.964
Missing	2 (28.6)	0	1 (14.3)	1 (33)	
Pathological response, (N=)	15	15	9	3	
Residual viable tumor					
<10%	7 (47)	1 (7)	6 (67)	1 (33)	
≥10%	8 (53)	14 (93)	3 (33)	2 (57)	0.018 *
Hyalinization/fibrosis					
>15%	6 (40)	4 (27)	5 (56)	2 (67)	
≤15%	6 (40)	11 (73)	4 (44)	1 (33)	0.373
Missing	3 (20)	0	0	0	

Values are n (%) unless otherwise indicated. Abbreviations: AS = angiosarcoma, LMS = leiomyosarcoma, Sarcoma NOS = Sarcoma not otherwise specified, SS = synovial sarcoma; N = number. * = *p* < 0.05; ^a^ = median and interquartile range is given, ^b^ = mean change in percentage and standard deviation is given.

**Table 3:** All areas under the curve (AUC) from the receiver operating characteristic curve analyses with corresponding optimal cutoff values *.

	Baseline PET Parameters			Evaluation PET Parameters		
	SUVmax	MTV	TLG	ΔSUVmax	ΔMTV	ΔTLG
Pathologic response, RVT						
AUC	0.501	0.719	0.704	0.807	0.642	0.621
Optimal CO value *	-	63	340	38%	-	-
Pathologic response, F/H						
AUC	0.532	0.706	0.684	0.865	0.748	0.748
Optimal CO value *	-	51	-	38%	60%	74%
Recurrence of disease						
AUC	0.523	0.796	0.758	0.652	0.736	0.711
Optimal CO value *	-	188	823	-	51%	50%

* Only displayed in the case of an AUC > 0.7. Abbreviations: AUC = area under the curve, CO = cutoff, RVT = residual viable tumor, F/H = fibrosis/hyalinization.

**Table 4:** Logistic regression analyses and Cox regression analysis with all established cutoff values for the PET parameters per corresponding outcome.

PET Parameter with Cutoff		N (%)	OR/HR	95% CI	*p=*
<10% Residual viable tumor					
MTV_BL_	≤63 mL	18 (43)	Ref		
	63 mL	24 (57)	0.21	0.05–0.82	0.024 *
TLG_BL_	≤340	19 (45)	Ref		
	>340	23 (55)	0.25	0.07–0.95	0.042 *
ΔSUVmax	≤38%	15 (54)	Ref		
	>38%	13 (46)	22.40	2.21–227.05	0.009 *
>15% Fibrosis/hyalinization					
MTV_BL_	≤51 mL	16 (38)	Ref		
	>51 mL	26 (62)	0.331	0.08–1.31	0.116
ΔSUVmax	≤38%	15 (54)	Ref		
	>38%	13 (46)	42.00	3.76–469.01	0.002 *
ΔMTV	≤60%	16 (67)	Ref		
	>60%	8 (33)	11.67	1.49–91.54	0.019 *
ΔTLG	≤74%	15 (63)	Ref		
	>74%	9 (37)	8.13	1.12–59.21	0.039 *
Recurrence of disease					
MTV_BL_	≤188 mL	18 (55)	Ref		
	>188 mL	15 (45)	5.11	1.40–18.67	0.014 *
TLG_BL_	≤823	17 (52)	Ref		
	>823	16 (48)	4.56	1.25–16.64	0.022 *
ΔMTV	≤51%	12 (50)	Ref		
	>51%	12 (50)	0.23	0.06–0.90	0.034 *
ΔTLG	≤50%	9 (38)	Ref		
	>50%	15 (62)	0.33	0.01–1.17	0.087

Abbreviations: N = Number, OR/HR = Odds ratio/Hazard ratio, CI = confidence interval. Odds ratios were calculated for pathological response and hazard ratios were calculated for oncological outcomes. * = *p* < 0.05.

## Data Availability

The raw data supporting the conclusions of this article will be made available by the authors upon request.

## Supplementary Materials

The following supporting information can be downloaded at: https://www.mdpi.com/article/10.3390/curroncol32050257/s1, Table S1: Patient, tumor, and treatment characteristics for patients with an early evaluation [^18^F]FDG PET/CT per histologic subtype.
