# Supplementary material for: The Role of [18F]FDG PET/CT Prior to and During Neoadjuvant Chemotherapy for Soft Tissue Sarcomas
Source: Curr Oncol. 2025 Apr 28;32(5):257. doi: 10.3390/curroncol32050257 (PMC12110720; doi:10.3390/curroncol32050257)
Supplement: Supplementary file 1 [file curroncol-32-00257-s001.zip › curroncol-3540477-supplementary.pdf]

Table S1. Patient, tumor, and treatment characteristics for patients with an early evaluation [^{18}F]FDG PET/CT per histologic subtype.

	All patients		AS		LMS		Sarcoma NOS		SS	
	N = 28		N = 7		N = 11		N= 7		N= 3	
Sex										
Male	10	(36)	2	(29)	3	(27)	3	(43)	2	(67)
Female	18	(64)	5	(71)	8	(73)	4	(57)	1	(33)
Age (IQR)	60	(49-63)	70	(39–74)	61	(52–63)	53	(50–59)	47	-
Stage										
Primary	23	(82)	5	(72)	9	(82)	6	(86)	3	(100)
Recurrence	4	(14)	1	(14)	2	(18)	1	(14)	0	-
Metastasis	1	(4)	1	(14)	0	-	0	0	0	-
FNCLCC grade										
1	4	(13)	0	-	4	(36)	0	-	0	-
2	8	(29)	0	-	5	(46)	1	(14)	0	-
3	8	(29)	2	(29)	2	(18)	6	(86)	0	-
Not graded[a]	8	(29)	5	(71)	0	-	0	-	3	(100)
Location										
Extremity	10	(36)	2	(29)	0	-	5	(72)	3	(100)
Mamma	4	(14)	4	(57)	0	-	0	-	0	-
Retroperitoneal	7	(25)	0	-	7	(64)	0	-	0	-
Abdomen	3	(11)	0	-	3	(9)	0	-	0	-
Trunk wall	2	(7)	0	-	1	(27)	1	(14)	0	-
Other	2	(7)	1	(14)	0	-	1	(14)	0	-
Preoperative RT										
Yes	7	(25)	1	(14)	1	(9)	3	(43)	2	(67)
No	21	(75)	6	(86)	10	(91)	4	(43)	1	(33)
Chemotherapy										
Paclitaxel	7	(36)	7	(100)	0	-	0	-	0	-
Dox/DTIC	10	(39)	0	-	10	(91)	0	-	0	-
Dox/ifos	11	(25)	0	-	1	(9)	7	(100)	3	(100)
N of cycles										
3	9	(32)	1	(14)	3	(27)	4	(57)	1	(33)
4	17	(60)	6	(86)	6	(55)	3	(43)	2	(67)
5	1	(4)	0	-	1	(9)	0	-	0	-
6	1	(4)	0	-	1	(9)	0	-	0	-

Values are n (%) unless otherwise indicated. Abbreviations: AS= angiosarcoma, LMS= leiomyosarcoma, Sarcoma NOS= Sarcoma not otherwise specified, SS= synovial sarcoma, N= Number, IQR= inter quartile range, Sarcoma NOS= sarcoma not otherwise specified, FNCLCC= Fédération Nationale des Centres de Lutte Contre le Cancer, RT= radiotherapy, NACT= neoadjuvant chemotherapy, Dox/ifos= doxorubicine/ifosfamide, Dox/DTIC= doxorubicine/dacarbazine,*= $p < 0.05$ TAC= docetaxel, doxorubicin, cyclophosphamide, a= pathologist in our center do normally not grade angiosarcoma and synovial sarcoma since they are always considered as high grade.
